# IFN-**α** blockade during ART-treated SIV infection lowers tissue vDNA, rescues immune function, and improves overall health

**DOI:** 10.1172/jci.insight.153046

**Published:** 2022-03-08

**Authors:** Louise A. Swainson, Ashish Arunkumar Sharma, Khader Ghneim, Susan Pereira Ribeiro, Peter Wilkinson, Richard M. Dunham, Rebecca G. Albright, Samson Wong, Jacob D. Estes, Michael Piatak, Steven G. Deeks, Peter W. Hunt, Rafick-Pierre Sekaly, Joseph M. McCune

**Affiliations:** 1 Division of Experimental Medicine, University of California, San Francisco, San Francisco, California, USA.; 2 Department of Pathology, Case Western Reserve University, Cleveland, Ohio, USA.; 3 Department of Pathology, Emory University, Atlanta, Georgia, USA.; 4 ViiV Healthcare, Research Triangle, North Carolina, USA.; 5 AIDS and Cancer Virus Program, Frederick National Laboratory for Cancer Research, Leidos Biomedical Research Inc., Frederick, Maryland, USA.; 6 Vaccine and Gene Therapy Institute and Oregon National Primate Research Center, Oregon Health & Science University, Portland, Oregon, USA.; 7 HIV Frontiers/Global Health Innovative Technology Solutions, Bill & Melinda Gates Foundation, Seattle, Washington, USA.

**Keywords:** AIDS/HIV, Immunology, Immunotherapy

## Abstract

Type I IFNs (TI-IFNs) drive immune effector functions during acute viral infections and regulate cell cycling and systemic metabolism. That said, chronic TI-IFN signaling in the context of HIV infection treated with antiretroviral therapy (ART) also facilitates viral persistence, in part by promoting immunosuppressive responses and CD8^+^ T cell exhaustion. To determine whether inhibition of IFN-α might provide benefit in the setting of chronic, ART-treated SIV infection of rhesus macaques, we administered an anti–IFN-α antibody followed by an analytical treatment interruption (ATI). IFN-α blockade was well-tolerated and associated with lower expression of TI-IFN–inducible genes (including those that are antiviral) and reduced tissue viral DNA (vDNA). The reduction in vDNA was further accompanied by higher innate proinflammatory plasma cytokines, expression of monocyte activation genes, IL-12–induced effector CD8^+^ T cell genes, increased heme/metabolic activity, and lower plasma TGF-β levels. Upon ATI, SIV-infected, ART-suppressed nonhuman primates treated with anti–IFN-α displayed lower levels of weight loss and improved erythroid function relative to untreated controls. Overall, these data demonstrated that IFN-α blockade during ART-treated SIV infection was safe and associated with the induction of immune/erythroid pathways that reduced viral persistence during ART while mitigating the weight loss and anemia that typically ensue after ART interruption.

## Introduction

Type I IFNs (TI-IFNs) are pleiotropic cytokines that serve as soluble mediators of response to viral infections and regulate the expression of genes associated with cell survival/death, proliferation, migration, and metabolism (1–3). Disruption of any of these axes can alter effector innate/adaptive immune responses and overall health. For example, expression of IFN-stimulated survival genes in effector T cells increases the efficacy of their responses while protecting them from NK cell–mediated killing (4, 5). On the other hand, TI-IFNs trigger death of memory T cells via proapoptotic cascades downstream of Bak/Bcl2-L-11 (6). TI-IFN disruption can thus alter the course of development of an effector immune response (7). During acute HIV infection, TI-IFN signaling induces restriction factors that inhibit HIV replication (8), and in vivo administration of IFN-α or IFN-β in rhesus macaques has been shown to prevent systemic SIV or SHIV infection (9, 10). Reciprocally, TI-IFN blockade during acute infection increases viremia, accelerating CD4^+^ T cell depletion and progression to AIDS (10, 11). Persistent TI-IFN signaling during chronic infection, on the other hand, may be detrimental: higher levels of TI-IFN signaling are correlated with immune activation (12, 13), poor immune reconstitution and blunted CD4^+^ T cell homeostasis with antiretroviral therapy (ART), and disease progression (14, 15). These negative effects are likely the result of many mechanisms, including cell death (16), upregulation of immunosuppressive pathways mediated by TGF-β, and CD8^+^ T cell exhaustion (17, 18). Thus, although TI-IFN may be beneficial during acute infection, sustained TI-IFN signaling can contribute to host-mediated immunopathology and disease progression.

This so-called “IFN paradox” (19) highlights the role of timing in the immunological effects of TI-IFN, a point underscored in 2 studies on chronic lymphocytic choriomeningitis virus (LCMV) infection. In a landmark series of experiments, the groups of Oldstone and Brooks showed that blockade of TI-IFN responses in LCMV infection leads to distinct outcomes that are dependent on the timing of the intervention (20). Specifically, TI-IFN inhibition early in infection was followed by decreased expression of antiviral IFN-stimulated genes (ISGs) and enhanced viral replication/dissemination. Sandler and Douek partially confirmed these findings in the nonhuman primate (NHP) SIV acute infection model, showing that administration of an antagonist to the TI-IFN receptor accelerates the course of infection and results in higher mortality rates (10). By contrast, TI-IFN inhibition during chronic LCMV disease diminished chronic immune activation while also restoring lymphoid tissue architecture and immune responses that ultimately facilitated viral clearance (21–24). The administration of anti–TI-IFN in chronic LCMV infection also led to downregulation of immune checkpoint proteins, improved CTL responses, and the reversal of disease progression (25). It is likely that alteration of pathways involved in the control of inflammation and immune homeostasis could facilitate viral clearance upon anti–TI-IFN treatment, e.g., by reduction in frequencies of TGF-β–secreting Tregs (26) (known to limit effector immune responses) and/or decreased death of memory T cells (6) (resulting in higher polyfunctional effector immune responses).

Pathogenic SIV infection of nonnatural host species (e.g., rhesus or pig-tailed macaques) is, like HIV infection, associated with robust TI-IFN responses that persist indefinitely (27); conversely, TI-IFN responses rapidly normalize after acute nonpathogenic SIV infection of the African green monkey and sooty mangabey (28–30). Underscoring the pathogenic role of persistent TI-IFN responses during HIV infection, blockade of TI-IFN signaling during chronic, ART-treated HIV infection of humanized mice has been observed to result in improved immune function, reduced HIV reservoirs, and delayed viral rebound during analytical treatment interruption (ATI) (31, 32). Though recent work in chronic, ART-treated SIV infection of rhesus macaques found that a TI-IFN receptor (IFNAR) antagonist that blocks the antiviral and antiproliferative activity of both IFN-α-2 and IFN-β (33) failed to modify plasma SIV RNA or cell-associated SIV DNA (34), we report here that IFN-α blockade (using an antibody that neutralizes 11 of 13 IFN-α isoforms) during ART-suppressed SIV infection of the rhesus macaque had a significant impact on SIV persistence and immune function as well as on clinical outcomes after interruption of ART.

## Results

**Figure 1:**
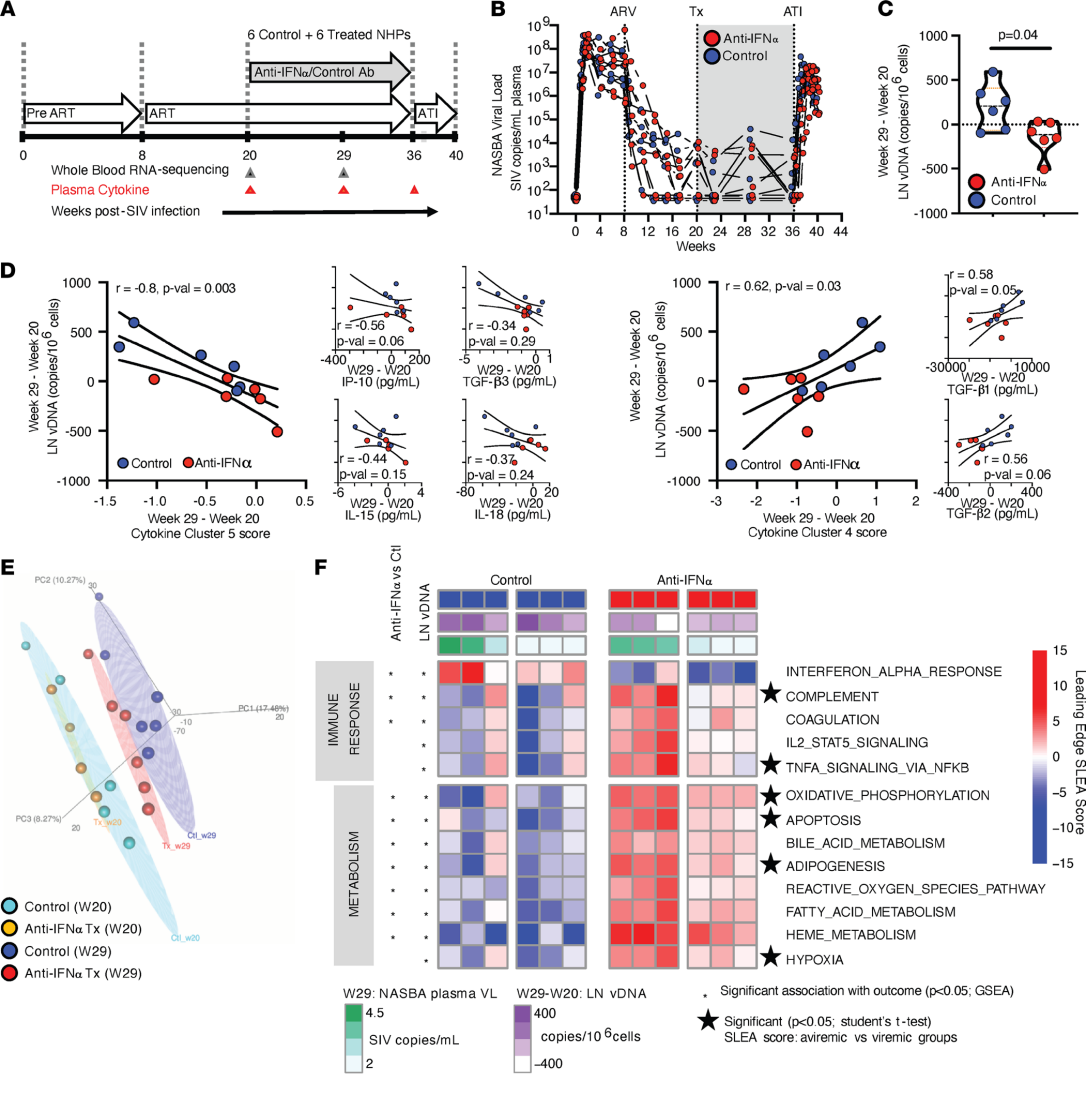
IFN-α blockade in ART-treated SIV-infected rhesus macaques causes a decline in LN vDNA, downregulation of TI-IFN signaling, and heightened proinflammatory cascades. (**A**) Study design showing the sampling schedule and timeline of ART, IFN-α blockade, and ATI after SIV infection. (**B**) Plasma SIV RNA levels measured during the study. (**C**) Decline in cell-associated LN vDNA (normalized to LN CD4^+^ T cell frequency) between weeks 20 and 29 in the anti–IFN-α-treated versus control arms (Mann-Whitney *U* test). (**D**) Plasma levels of cytokines measured and the composition of circulating cytokines defined by clustering cytokines using k-means clustering. The decline in LN vDNA was associated with increases in cytokine cluster 5 (left) and decreases in cytokine cluster 4 (right). The association with cytokines that define clusters 4 and 5 is also shown. All correlations with decline in LN vDNA were assessed using Spearman’s correlation test. (**E**) Principal component analysis (PCA) of whole-blood transcriptome profiles performed prior to initiating IFN-α blockade (week 20) and 9 weeks after blockade (week 29). Anti–IFN-α-treated samples observed to cluster distinctly from the control arm at week 29. (**F**) Heatmap showing the sample-level leading-edge scores of immune/metabolic gene sets (MSigDB’s Hallmark module; ref. 40) and cell subset signatures (44) significantly altered with anti–IFN-α treatment (vs. control arm; week 29) and/or associated with decline in LN vDNA (column annotations in purple) (Supplemental Table 3). GSEA (38) was used to determine significance with each outcome, and significant associations are shown as *P < 0.05. Sample-level scores (75) intersecting leading-edge genes (obtained after GSEA results) between virally suppressed and incompletely suppressed NHPs in both arms (3 NHPs per group per arm). Stars define significance (assessed using a 2-tailed unpaired *t* test) between the 2 groups with *P* < 0.05. No significant differences between virally suppressed and incompletely suppressed NHPs were observed in the untreated arm. Unless indicated, significant changes in NHP specimens between the 2 arms (*n* = 6/arm) 29 weeks after study initiation were assessed using the statistical tests indicated.

We assessed the impact of broad IFN-α signaling blockade in SIV-infected rhesus macaques that had been suppressed on ART for 12 weeks. We hypothesized that TI-IFN inhibition would lead to enhanced effector immune responses and a reduction in the viral reservoir. AGS-009, an antibody that neutralizes 11 of the 13 IFN-α isoforms (11, 35), was administered weekly for 16 weeks, followed by a 4-week ATI. Clinical, immunological, and molecular outcomes were assessed throughout the study (Figure 1A). Animals undergoing IFN-α blockade showed no abnormal clinical signs or behavior, and labs for complete blood counts and routine chemistries were normal (Supplemental Table 1; supplemental material available online with this article; https://doi.org/10.1172/jci.insight.153046DS1). Although plasma SIV RNA levels were comparable in the treated and control groups throughout the study, with incomplete viral suppression in some animals from both groups (Figure 1B), IFN-α blockade resulted in a significant decline in lymph node (LN) viral DNA (vDNA) at 9 weeks relative to treatment with control antibody (*P* = 0.04) (Figure 1C and Supplemental Figure 1A). It should be noted that no significant association in LN vDNA levels and incomplete viral suppression was observed, suggesting that the decline in LN vDNA after treatment was primarily driven by IFN-α blockade (Supplemental Figure 1B).

### TI-IFN blockade induces a pleiotropic shift in systemic plasma and cellular profiles that is associated with a decline in LN vDNA.

Since several TI-IFN–regulated proinflammatory cytokines (e.g., IL-6 and TNF-α) have been previously associated with HIV reservoir size (36), we used MesoScale Discovery’s electrochemiluminescence U-plex platform to quantify changes in plasma levels of 23 cytokines after IFN-α blockade (See Supplemental Table 1 for raw plasma cytokine concentrations). These data were binned into 6 optimal cytokine clusters, defined as homogenous groups of cytokines and determined by applying k-means clustering and gap-statistics analyses (37) using the “cluster” and “factoextra” packages in R (Supplemental Table 2: List of all cytokine cluster members and cluster centroid scores). Cluster 5 cytokines (including proinflammatory cytokines, IP-10, IL-15, and IL-18, known to enhance innate and cell-mediated immune responses) increased with IFN-α blockade in association with a decrease in LN vDNA (ρ = –0.80, *P* = 0.003) (Figure 1D). In contrast, those in cluster 4 (including antiinflammatory cytokines, TGF-β1 and TGF-β2, known to inhibit effector T cell differentiation) decreased with IFN-α blockade in association with a decrease in LN vDNA (ρ = 0.62, *P* = 0.03) (Figure 1D). These results indicate that blockade of IFN-α during ART resulted in a decrease in LN vDNA that was associated with increased proinflammatory cytokines and decreased antiinflammatory cytokines, changes that would predictably enhance cell-mediated immunity.

**Figure 2:**
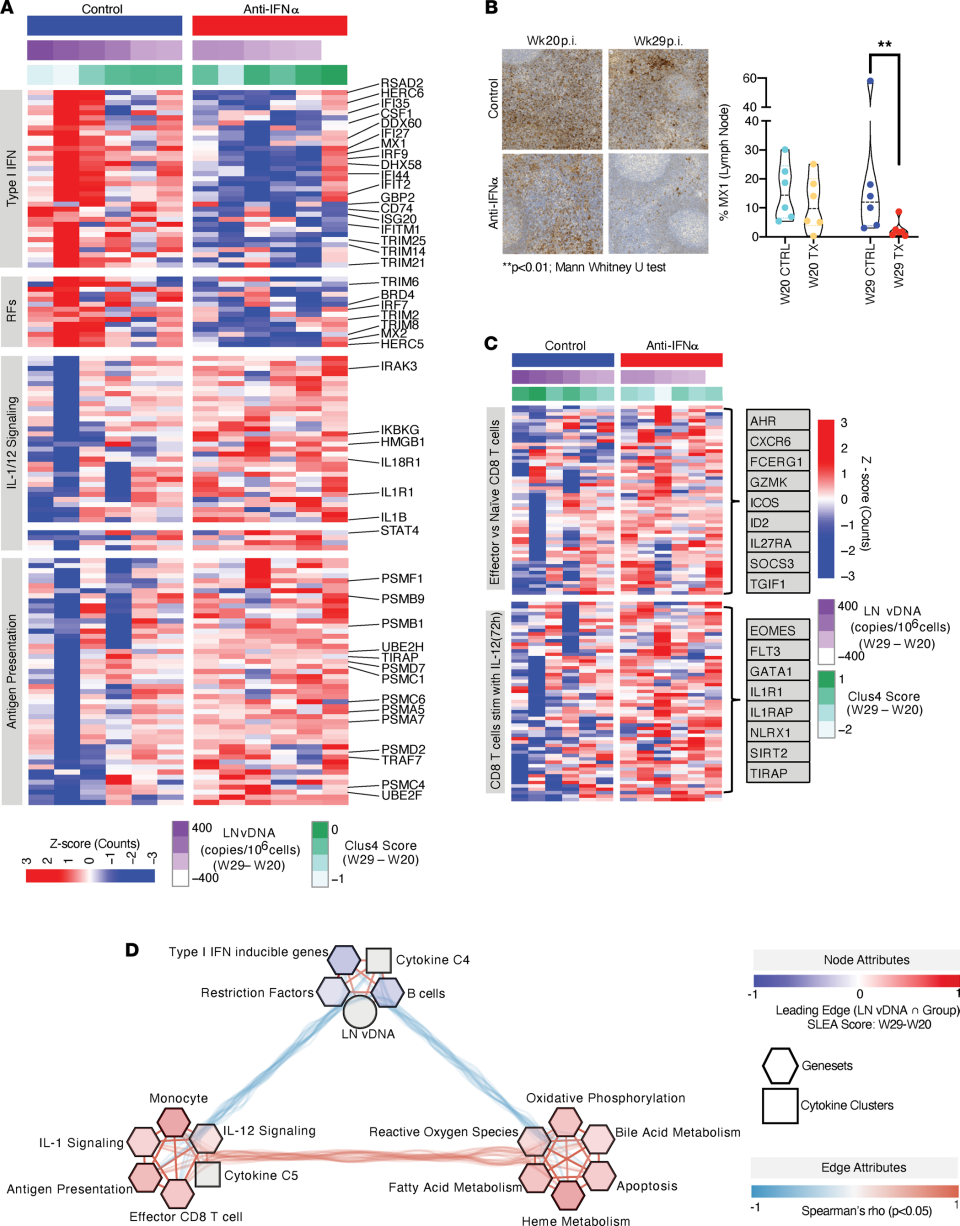
IFN-α blockade leads to a downregulation of innate antiviral genes, plasma TGF-β levels, and TI-IFN–inducible gene sets and upregulation of antigen presentation, IL-1, IL-12 signaling cascades, and effector CD8^+^ T cell signatures. (**A**) Heatmap showing leading-edge genes of immune genesets (40,43) that are significantly associated with reduction in LN vDNA (GSEA results in Supplemental Table 4; *P* < 0.05) and increase in cytokine cluster 5. (**B**) In conjunction with the systemic downregulation of antiviral genes with anti–IFN-α treatment, immunohistochemistry staining shows the downregulation of the prototypical antiviral protein, Mx1, in lymph nodes 9 weeks after anti–IFN-α (vs. control arm; Mann-Whitney *U* test, **P < 0.01). (**C**) Leading-edge genes from 2 effector CD8^+^ T cell signatures (GSE40666: defining effector vs. naive CD8^+^ T cell expression signatures and GSE15930: defining CD8^+^ T cell signatures induced after IL-12 treatment in vitro) were enriched after IFN-α blockade and associated with a decline in LN vDNA and a decrease in antiinflammatory cluster 4 cytokine levels (Supplemental Table 6). (**D**) Correlation network between nodes that represent change in LN vDNA, cytokine clusters, and SLEA (75) scores of intersecting leading-edge genes resulting from GSEA (38) against the 2 main outcomes (i.e., group comparison anti–IFN-α vs. control and change in LN vDNA). Node colors (in all nodes except LN vDNA and cytokine scores) represent change in SLEA scores between anti–IFN-α treatment and control groups at week 29 (red and blue represent increase and decrease in anti–IFN-α-treated groups, respectively). Edges represent correlations (ρ values calculated after Spearman’s test; edges with *P* < 0.05 shown) between nodes, where edges directly connected with LN vDNA are thickened to show direct correlates of the outcome of interest. Unless indicated, significant changes in NHP specimens between the 2 arms (*n* = 6/arm) 29 weeks after study initiation were assessed using the statistical tests indicated.

To further explore mechanisms driving the reduction in LN vDNA, we performed RNA-Seq on whole-blood samples obtained before and after IFN-α blockade. Preblockade, animals in each group showed similar gene expression profiles by principal component analysis (PCA). Nine weeks after IFN-α blockade, those in the treatment group had gene expression profiles that were distinct from those in the control arm (Figure 1E). Gene set enrichment analyses (GSEA; ref. 38) were applied to define the immune, metabolic, and cell cycling pathways (gene sets extracted from MSigDB’s Hallmark module; refs. 39, 40) that were modulated by IFN-α blockade and were associated with the reduction in LN vDNA (Supplemental Table 3: GSEA results with normalized enrichment score (NES) values, *P* values, leading-edge genes common to both outcomes). As expected, IFN-α blockade resulted in downregulation of genes that are known targets of IFN-α (ISGs) (Figure 1F), e.g., transcription factors like IRF-7 and IRF-9 that further promote TI-IFN responses (41, 42) (Supplemental Figure 1C) and genes known to restrict HIV replication (e.g., *MX1*, *MX2*, *RSAD2*, *CD74*, and *BRD4*; ref. 43) (Figure 2A; Supplemental Table 4: Statistics and full list of leading-edge genes in Figure 2A). Downregulation of antiviral Mx1 protein expression was verified by immunohistochemistry of LN sections 9 weeks after anti–IFN-α treatment (Figure 2B). The reduced TI-IFN signature observed even after 9 infusions of the ASG-009 antibody (9 weeks after treatment initiation) suggests a lack of an anti-drug antibody effect. LN vDNA reduction after IFN-α blockade was also associated with decreased gene expression pathways linked to plasmacytoid DCs (pDCs) and B cells (Supplemental Figure 1D; Supplemental Table 3; ref. 44). Interestingly, these downregulated signatures (in addition to being inclusive of typical B cell genes like *CD22* and *CD79A*) included genes that restrict effector T cell responses like the IL-18 blocker *IL18BP*. Inhibition of this gene has recently been shown to promote polyfunctional CD8^+^ T cell responses (45) (Supplemental Figure 2, A and B). The downregulation of such genes after anti–IFN-α treatment supports the observed increase in levels of proinflammatory cytokine cluster 5 (composed of IP-10, IL-15, and IL-18). Together, these observations showed that the decrease in LN vDNA observed after IFN-α blockade was associated with upregulation of a cluster of proinflammatory plasma cytokines and occurred despite downregulation of ISGs and restriction factors known to inhibit HIV replication.

Consistent with the pleiotropic effects of TI-IFNs, the decline in LN vDNA after IFN-α blockade was also associated with a significant increase in metabolic gene sets, including those associated with fatty acid metabolism/adipogenesis (e.g., *CPT2*; ref. 46), oxidative phosphorylation (e.g., *NDUFs* and *COXs*; refs. 39, 40, 47), innate proinflammatory responses (e.g., TNF-α/NF-κB cascade, complement, and coagulation cascades), and adaptive effector T cell responses (e.g., STAT5A signaling downstream of IL-2) (Figure 1F). It should be noted that, although all NHPs receiving IFN-α blockade upregulated these gene sets, the expression was significantly higher in animals that showed incomplete viral suppression with ART (Figure 1F). Overall, increased expression of metabolic/proinflammatory genes in the NHPs receiving IFN-α blockade was complemented by increases in gene signatures specific to monocytes and myeloid DCs (mDCs), with leading-edge genes characterizing the innate immune signatures inclusive of components of the IL1R complex *IRAK3* (kinase downstream of IL1R), *NLRP3*, and *CASP1* (required for IL-1β activation and effector function) (48) (Supplemental Figure 2, C and D). To confirm that the systemic changes induced by this treatment were also observed in HIV-harboring tissues, we performed RNA-Seq on rectal biopsies from all NHPs before and 9 weeks after IFN-α blockade. We observed that, as in the case of whole blood, the IFN-α response gene set (which included the aforementioned genes, *MX1* and *IRF7*) was also significantly reduced in rectal biopsies (GSEA *P* < 0.05); concomitantly, the gene set regulated by TNF-α/NF-κB cascade (including members of the c-Jun complex) was significantly induced 9 weeks after treatment (GSEA *P* < 0.05) (Supplemental Figure 2E). Detailed analyses of innate proinflammatory gene sets that are associated with the decline in LN vDNA after IFN-α blockade and proinflammatory cluster 5 levels revealed that the expression of genes in the IL-1/18 signaling cascade (including *IL18R1*, *IKBKG*, and *MAPKs*) (Figure 2A and Supplemental Table 4) and expression of target genes of NF-κB p65 (master regulator of innate immune responses and subunit of NF-κB transcription factor complex) were significantly increased (Supplemental Figure 3, A–C; Supplemental Table 5: Statistics and full list of leading-edge genes in Supplemental Figure 3A). These data demonstrated that a distinct inflammatory state triggered by IFN-α blockade drove lower tissue vDNA, despite downregulation of restriction factors known to inhibit HIV replication.

### The decline in LN vDNA after TI-IFN blockade is associated with enhanced gene expression profiles defining antigen presentation, IL-12 signaling, and effector CD8^+^ T cell responses.

Given that TI-IFN signaling in chronic viral infection has been associated with higher regulatory T cell activity (17) and lower CD8^+^ T cell responses (18), we investigated the impact of IFN-α blockade on molecular and cellular gene expression profiles defining effector T cell responses. The decline in LN vDNA after IFN-α blockade was associated with expression of genes important for proteasomal antigen presentation, cell-mediated immunity via IL-12 signaling (as characterized by increased expression of *STAT4*; ref. 49) (Figure 2A) (Supplemental Table 4), and transcription factor target (TFT) genes (ChIP-Seq validated TFTs extracted from the CHEA database; ref. 50) downstream of the transcription factors STAT5A (driver of T cell proliferation and differentiation; ref. 51) and RAR-γ (master regulator of T helper 17 cell differentiation; ref. 52) (Supplemental Figure 3, A and B; Supplemental Table 5). These enhanced expression profiles were reciprocated by reduced expression of target genes downstream of transcription factors driving quiescence/stemness (e.g., TCF-7, β-catenin, and FOXM1; refs. 53, 54) (Supplemental Figure 3, A and B; Supplemental Table 5). Integration of the TFT expression profile with cytokine expression data revealed the following: (a) higher levels of the proinflammatory cytokine cluster 5 (an inverse correlate of LN vDNA decline) were associated with STAT5A target genes, and (b) lower levels of the antiinflammatory cluster 4 (composed of TGF-β1/TGF-β2 and a direct correlate of LN vDNA decline) were associated with decreased expression of TCF-7 targets. (Supplemental Figure 3C). These data suggest that the decline in LN vDNA levels observed after IFN-α blockade could result from enhanced effector CD8^+^ T cell responses.

Although sufficient viably cryopreserved PBMCs were not available to directly assess changes in SIV-specific CD8^+^ T cell responses, we assessed whether publicly available effector CD8^+^ T cell signatures were enriched after IFN-α blockade (55, 56). This analysis revealed that effector but not naive CD8^+^ T cell gene signatures were significantly associated with both IFN-α blockade and the decrease in LN vDNA (Figure 2C; Supplemental Table 6: Statistics and full list of leading-edge genes in Figure 2C). These signatures were exemplified by increased expression of genes, such as *ID2* and *AHR*, known to drive effector T cell differentiation (57, 58) (Figure 2C) and increased expression signatures characteristic of CD8^+^ T cells stimulated in vitro with IL-12 (59) (a prototypic Th1 cytokine) (Figure 2C). These data suggest that reduced LN vDNA after IFN-α blockade might result from the rescue of IL-12/STAT4-dependent Th1 and effector CD8^+^ T cell responses. Integrated modeling also revealed that changes in effector CD8^+^ T cell signatures after IFN-α blockade were associated with decreased expression of TFTs that drive T cell quiescence/stemness (e.g., *TCF7* and *CTNNB1*; ref. 53) (Supplemental Figure 3D) and increased expression of transcription factors/TFTs that drive T cell proliferation (e.g., STAT5A targets; ref. 51) and differentiation (e.g., *VDR*, *ID2*, and *EOMES*; refs. 58, 60, 61) (Supplemental Figure 3, D and E). These effector CD8^+^ T cell signatures correlated with IL-12 and antigen presentation signaling signatures as well as heightened metabolic signatures (Figure 2D). Overall, these observations showed that the decline in LN vDNA seen after IFN-α blockade was associated with reduced plasma TGF-βs, with concomitant increases in monocyte signatures and cytokine signaling cascades downstream of IL-1s and IL-12 pathways that support Th1 immunity and cytotoxic T cell differentiation (Figure 2D).

### ATI after IFN-α blockade is associated with lower levels of weight loss and improved erythroid function.

**Figure 3:**
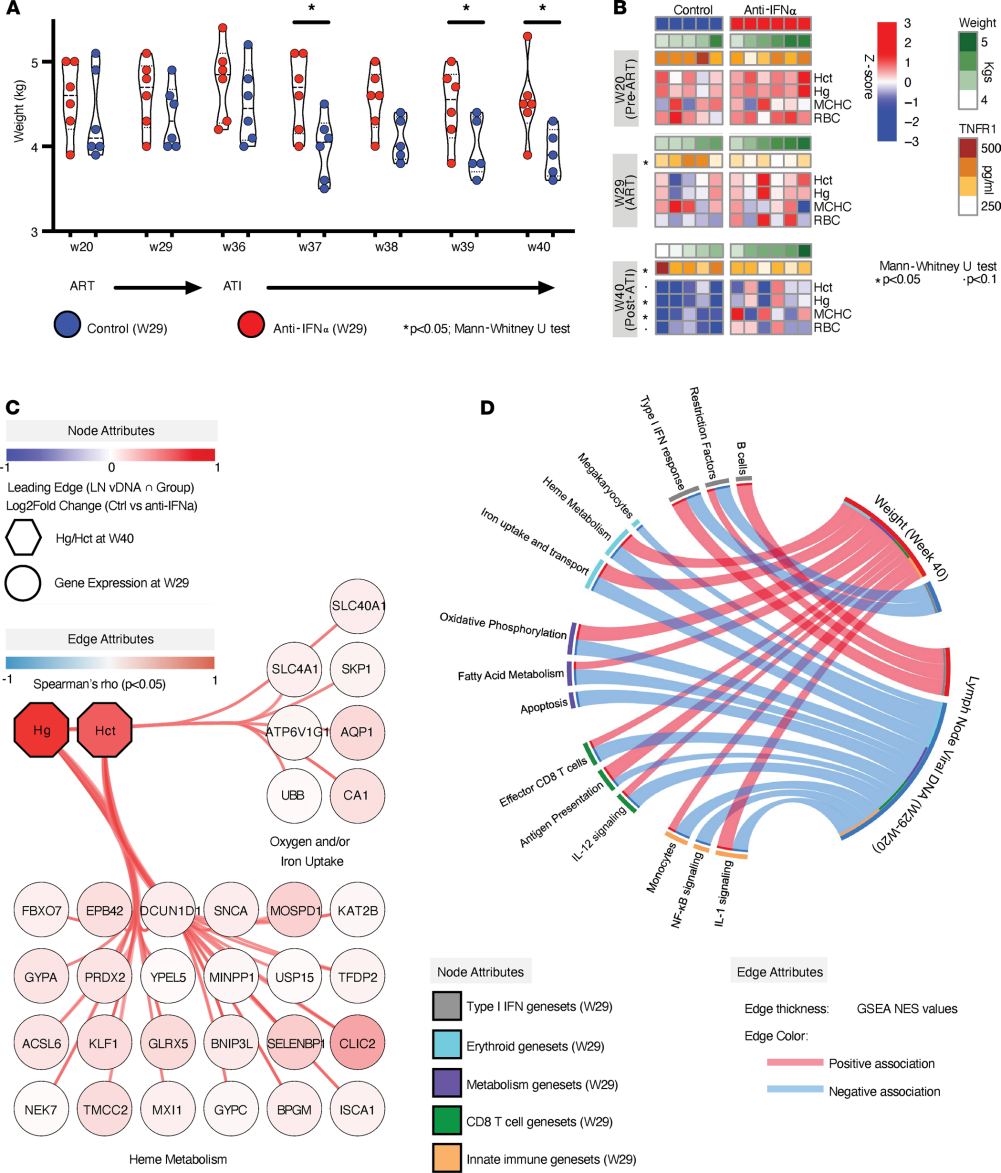
Heightened expression of metabolic, erythroid, and proinflammatory gene sets during ART are associated with maintenance of weight after ATI in anti–IFN-α-treated rhesus macaques. (**A**) As monitored after ATI (week 36 to week 40), weight was found to be maintained in NHPs in the anti–IFN-α-treated arm compared with those in the control arm (Mann-Whitney *U* test per week; *P < 0.05). (**B**) Assessment of physiological outcomes (analogous to the components of VACS index; ref. 64) before and after IFN-α blockade and after ATI revealed that levels of sTNFR1 and erythroid function were differentially altered (Mann-Whitney *U* test; *P < 0.05, •*P* < 0.1 (See Supplemental Table 1 for full list of physiological markers). Only 5 control animals are represented after ATI because 1 animal from this group died during the ATI period. (**C**) Correlation network highlighting the association with levels of hemoglobin and hematocrit (at week 40 after infection; i.e., after ATI) with gene expression of heme metabolism and iron/oxygen pathways (29 weeks after infection; during ART). The node colors indicate log_2_ (fold change) between anti–IFN-α-treated and control arms (where red to blue colors reflect high to low fold change), whereas the edge colors indicate Spearman’s correlation coefficients between the nodes (where red to blue colors reflect high to low correlations). (**D**) Circos plot with edges representing NES values (GSEA *P* < 0.05; positive NES values shown in red; negative NES values shown in blue) defining associations between key gene sets (altered after anti–IFN-α treatment) and major outcomes: weight maintenance after ATI and decline in LN vDNA (Supplemental Table 8). Unless indicated, significant changes in NHP specimens between the 2 arms (*n* = 5 control arm and *n* = 6 treatment arm) at all time points after study initiation were assessed using the statistical tests indicated.

As cachexia (with extreme weight loss and muscle wasting) is a hallmark of AIDS (62) and TI-IFNs are known to induce TNF-α/cachectin, we assessed the impact of IFN-α blockade on body weight. Before and during ART, the weight of animals infused with anti–IFN-α was not significantly different from that of control animals. However, upon ATI, animals in the anti–IFN-α-treated arm were observed to maintain weight at significantly higher levels compared with those in the control arm (Figure 3A). Consistent with previous reports linking soluble plasma TNF-α receptor (sTNFR1) levels with cachexia (63), sTNFR1 levels in animals treated with anti–IFN-α were significantly reduced before and after ATI (Figure 3B). To better understand the physiological parameters responsible for such maintenance of weight after IFN-α blockade, we also assessed post-ATI alterations in the clinical variables of the VACS index, an index of multiorgan clinical variables, including measures of erythroid, renal, liver, and metabolic function (Supplemental Table 1 and Supplemental Table 7: List of VACS variables), which predict mortality in HIV infection (64). We observed that RBC counts and other markers of erythroid function, e.g., hemoglobin (Hg), mean corpuscular hemoglobin concentration (MCHC), and hematocrit (Hct), were significantly reduced in the control group after ATI, whereas the anti–IFN-α–treated animals maintained these markers at similar levels after ATI (Figure 3B). The increase in Hg and Hct at week 40 during IFN-α blockade was driven by an increase in expression of pathways associated with iron/oxygen uptake (characterized by an increase in aquaporin 1 and iron transporter, ferroportin [*SLC40A1*]) and heme metabolism (including master regulator of hematopoiesis/erythropoiesis, *KLF1*; ref. 65) (Figure 3C). In line with these data, maintenance of weight after ATI was associated with greater expression of gene sets related to metabolism (e.g., for fatty acid and bile metabolism and oxidative phosphorylation) and erythropoiesis (e.g., heme metabolism, GATA-1/Nrf-2 target expression) during the time of anti–IFN-α treatment (Figure 3D and Supplemental Table 8).

In sum, IFN-α blockade caused a decline in LN vDNA during ART and maintenance of weight when ART was discontinued. These events were associated with the induction of gene expression modules that encompass increases in erythropoiesis, mitochondrial function, and fatty acid metabolism (Figure 3D). Such an enhanced systemic metabolism could play a crucial role in supporting innate proinflammatory responses (i.e., IL-1 signaling in monocytes and IL-12 signaling) and effector CD8^+^ T cell responses that drive the decline in LN vDNA levels, despite reduced expression of ISGs/antiviral genes after IFN-α treatment (Figure 3D). Concurrently, the increase in systemic metabolism and erythrocyte function observed after anti–IFN-α treatment may mitigate weight loss and anemia that typically ensue after ART interruption (66) (Figure 3D).

## Discussion

Humanized mouse studies (31, 32) and this rhesus macaque study demonstrated that, in the setting of chronic ART-treated HIV/SIV infection, sustained TI-IFN signaling may contribute to immune dysfunction and foster SIV/HIV persistence, and that blockade of IFN-α can provide clinical benefit. These results differ from those of Douek and colleagues, who did not observe a significant effect of TI-IFN receptor antagonism on plasma SIV RNA or on circulating or LN cell–associated SIV DNA levels in ART-treated, chronic SIV–infected rhesus macaques (34). Unlike the TI-IFN receptor antagonist used in the latter study, the AGS-009 antibody in this study had a longer half-life in vivo (~8 days vs. 19 hours) and could be used at fewer repeated infusions (1 per week vs. 2–3 per week) while continuing to maintain a repressed IFN-inducible gene expression profile 9 weeks after treatment (Figure 2A). These data are in line with work in cynomolgus macaques, where the repeated use of AGS-009 resulted in persistent suppression of TI-IFN signaling and was not associated with a T-dependent antibody response (TDAR) after 13 weekly infusions (data not shown). In our study, the suppression of TI-IFN signaling after 9 weekly doses was associated with a decline in LN vDNA levels, suggesting that longer-term IFN-α blockade might prove to be even more effective in the reduction of SIV DNA levels in reservoir tissues. Finally, although we did not observe a significant decline in plasma viremia upon interruption of ART, physiological readouts (such as maintenance of weight and erythrocyte function) in NHPs receiving AGS-009 demonstrated that the use of this antibody could mitigate the adverse health effects of ART interruption. Together, our data demonstrated that suppression of TI-IFN signaling in ART-suppressed NHPs using an antibody like AGS-009 was both safe and capable of initiating cellular/molecular mechanisms that serve to reduce LN vDNA levels in reservoir sites like LNs, while also providing clinical benefit upon interruption of ART.

To explore the mechanisms that drive IFN-α blockade–associated declines in LN vDNA, our study evaluated the expression of plasma cytokines and of the whole blood transcriptome. We observed that the decline in LN vDNA occurred despite a reduction in TI-IFN–induced genes and innate restriction antiviral factors. More importantly, IFN-α blockade initiated a cascade of immunological events that encompassed processes in both the innate and adaptive arms. NHPs receiving IFN-α blockade were, by example, observed to have higher expression of myeloid (mDC and monocyte) gene sets that would be expected during an early proinflammatory innate immune response (e.g., higher IL-1β and IL-12 signaling). In line with previous studies showing a role of TI-IFNs in restricting IL-12 signaling (67), our data demonstrated a rescue of innate immune function accompanied by increased expression of antigen presentation and IL-12–induced effector CD8^+^ T cell gene sets. An increase in plasma cytokines supportive of heightened cytotoxic cell migration and activation (e.g., IL-15, IP-10, and IL-18) would also be consistent with an increase in effector responses. A significant decrease in plasma TGF-β (likely resulting from an M1-like polarization of the innate compartment or lower Treg activity) would conceivably also contribute to augmented cytotoxic antiviral responses. Importantly, these effects of IFN-α blockade on putative effector responses appeared to be most dramatic among macaques with incomplete plasma virus suppression, suggesting that the suppressive effect of IFN-α on effector responses is most relevant in the context of chronic antigen exposure. Overall, the restoration of CD8 effector function in anti–IFN-α-treated animals together with residual low-level viremia and presentation of viral antigens to CD8^+^ T cells could result in the killing of infected cells and decrease in LN vDNA. Although we were unable to directly assess SIV-specific T cell frequency and function because of limited recovery of viable cryopreserved PBMCs, we observed that restoration of CD8^+^ T cell effector function signature was supported by prior studies of TI-IFN blockade in humanized mice where HIV-specific CD8^+^ T cell function was rescued (31, 32).

Aside from initiating antiviral immune responses that could help purge the HIV reservoir in the long run, we observed that anti–IFN-α treatment helped mitigate the adverse health effects of treatment interruption, including anemia and weight loss. To characterize overall health status of NHPs in this study, we used the VACS index variables (including several indices of multiorgan function that predict mortality in people with HIV) to determine whether anti–IFN-α treatment had a significant impact on overall health during ATI. Our data showed that anti–IFN-α-treated NHPs were able to maintain their weight up to 4 weeks after ART. These data confirmed the role of TI-IFN signaling pathway observed in a previous study that used the LCMV model to show that T cell–intrinsic TI-IFN signaling pathway can lead to infection-associated cachexia (68). In line with reduced cachexia, our data showed that sTNFR1 was reduced before and after ART in the treatment arm. More importantly, we observed that expression of pathways that drive erythropoiesis, heme metabolism, and iron uptake were enriched in the anti–IFN-α treatment arm and culminated in maintenance of RBC counts and Hg levels upon ATI. Overall, these data indicated that IFN-α blockade activated transcriptional pathways that preserve RBC production, iron/oxygen uptake, and an overall metabolic activity, thereby mitigating the adverse health effects of ART interruption (66), despite lack of viral control.

This study is the first of its kind to our knowledge to use an integrated “-omics” approach to demonstrate that pathways downstream of IFN-α blockade encompassing enhanced innate immune activation, adaptive effector responses, and erythroid function regulate HIV reservoir size during ART, mitigating weight loss and anemia during ATI. Specifically, our data highlighted the resurgence of monocyte activation and IL-12–dependent effector CD8^+^ T cell responses when TI-IFN responses were blocked. Concurrently, heightened oxygen/iron uptake along with expression of heme (known to drive better physiological outcomes) and other metabolic pathways was evident. Taken together, these findings suggest that broad IFN-α blockade in the context of chronic, ART-suppressed HIV infection is safe and may lead to improved CD8^+^ T cell cytotoxic function and viral reservoir reduction during ART and better clinical outcomes upon interruption of ART.

## Methods

### Study design.

Twelve female rhesus macaques (4–5 years of age) were i.v. challenged with SIVmac251 after testing negative for simian retrovirus, simian T lymphotropic virus, hepatitis B, and helminthic infections. The viral stock was previously characterized and obtained from (data not shown) Nancy Miller, National Institutes of Health, Bethesda, Maryland, USA). At 8 weeks after infection, animals commenced an ART treatment regimen of L-612 (RAL, 150 mg [po, bid], Merck), emtricitabine (FTC, 30 mg/kg/day, Gilead), tenofovir (PMPA, 20 mg/kg/day, Gilead), and darunavir (DRV, 400 mg [po, bid], Tibotec) until ATI at 36 weeks after infection After 12 weeks of ART (20 weeks after infection), 6 animals received anti–IFN-α humanized antibody AGS-009, a gift from Argos Therapeutics i.v. at 10–11 mg/kg, and the other 6 control animals received an irrelevant antibody (an anti–hepatitis B antibody, prepared by Keith Reimann, formerly of Mass Biologics/UMass Chan Medical School) weekly for 16 weeks, a regimen guided by previous work in humans and in cynomolgus macaques (11, 69). AGS-009 and control antibody were i.v. administered in 50 mL saline solution with an injection rate of 2.5–8 mL/min. An additional 10 mL of saline was used to flush the syringe, leading to a total of 60 mL saline and drug injected. At 36 weeks after infection, all animals were taken off both ART and treatment with either anti–IFN-α or control Ab for 7 weeks before necropsy. Peripheral blood, rectal pinch biopsies, and peripheral LN biopsies were sampled to evaluate viral load, CD4^+^ T cell counts, immune phenotype and function, and transcriptional and serological signatures. The study design and schedule are shown in Figure 1A.

### ELISAs.

Plasma was stored at –80°C prior to analysis. sTNFR1 was measured using the R&D Systems ELISA kit for sCD14 and IP-10 according to the manufacturer’s instructions.

### Immunohistochemistry.

Immunohistochemical staining and quantitative image analysis were performed as previously described (70). In brief, immunohistochemistry was performed using a biotin-free polymer approach (Golden Bridge International) on 5 μm tissue sections mounted on glass slides, which were dewaxed and rehydrated with double-distilled water. Heat-induced epitope retrieval was performed by heating sections in 0.01% citraconic anhydride containing 0.05% Tween 20 for Mx1 antibody. Slides were incubated for 1 hour at room temperature with mouse anti-Mx1 mAb (1:2000; a gift from Georg Kochs and the Department of Virology of the University of Freiburg, Germany, clone M143) diluted in blocking buffer. After washing in 1× TBS with 0.05% Tween 20, endogenous peroxidases were blocked using 1.5% (v/v) H_2_O_2_ in TBS, pH 7.4, for 5 minutes, and the slides were incubated with mouse Polink 1 (Golden Bridge International) HRP and developed with ImmPACT DAB (Vector Laboratories), according to the manufacturer’s recommendations. All slides were washed in tap water, counterstained with hematoxylin, mounted in Permount (Thermo Fisher Scientific), and scanned at high magnification (×200) using the ScanScope AT2 System (Aperio Technologies), yielding high-resolution data from the entire tissue section. Representative regions of interest (500 × 500 μm) were identified, and high-resolution images were extracted from these whole-tissue scans. The percentage area of the positive cell zone was quantified using Photoshop CS5 and Fovea tools.

### Inguinal LN and rectal viral load DNA assessment.

Tissue-associated SIV RNA and DNA levels were determined as described (71, 72), and then normalized to CD4^+^ T cell frequency in the tissue as determined by flow cytometry (See Supplemental Table 1 for values).

### Preprocessing of RNA-Seq data from whole blood and rectal biopsies.

At weeks 20 and 29, 10 mL of whole peripheral blood (collected in PAXgene tubes) and rectal biopsies were collected and cryopreserved at –80°C. The PreAnalytix PAXgene Blood RNA or Qiagen’s RNeasy kits were used for RNA extraction, and an RNA-integrity number of 8 or above (measured using a bioanalyzer) was confirmed. Library preparation was performed using the TruSeq Stranded Total RNA Library Prep kit after rRNA and globin mRNA removal using the Globin-Zero Gold rRNA removal kit. Sequencing was performed using the HiSeq 2500 (Illumina Inc.), with approximately 30 million paired-end reads obtained per sample. The sequencing FASTQ files were aligned to the Macca Mulatta (RefSeq Mmul_10) using the rapid Spliced Transcripts Alignment to a Reference (STAR) Aligner (73). Exonic transcript abundance of mapped reads was determined using HTSeq, and the final counts were normalized by TMM (trimmed mean of M values) by correcting for library size. Raw FASTQ files and processed/normalized data for all samples included in this study can be accessed in NCBI’s Gene Expression Omnibus (GEO GSE184495).

### Plasma cytokine collection and initial data processing.

Custom U-plex plates developed by the MesoScale Discovery team were used to assess the levels of 19 detectable (of the 23 measured) cytokines in the plasma of all animals at weeks 20, 29, and 36: IL-17α, IL-1β, IL-2, IL-4, IL-6, IL-8, IL-9, IP-10, TNF-α, TGF-β1, TGF-β2, TGF-β3, IFN-α2α, IL-10, IL-15, IL-16, IL-18, IL-22, and IL-7. TGF-β1/2/3 were run in 1 plate; the plasma was acid-treated and base-neutralized to activate the TGF family proteins prior to execution of the standard Mesoscale Discovery Soluble protein assessment protocols using the U-plex kit. Cytokine levels were measured from 25 μL of undiluted plasma in duplicate wells. Plates were run on the Meso Scale Discovery instrument (Meso Scale Discovery) and cytokine levels calculated using a standard curve of known cytokine quantities. Nineteen of the 23 cytokines measured were detected in more than 50% of the samples and showed low coefficient of variation between duplicates (<20%) (See Supplemental Table 1 for plasma cytokine concentrations at weeks 20, 29, and 36 after infection). The optimal number of clusters for all detectable cytokines in week 29 samples was determined using the clusGap function in R (Package: Cluster), where the clustering function was set to k-means. The optimal number of clusters was calculated by assessing the gap-statistic (37) using the first SEmax method using the factoextra package. K-means clustering and cluster center calculations were done on the optimal number of clusters using the k-means function (Supplemental Table 2).

### Multiomic data analyses, statistics, and integration of RNA-Seq data.

PCA on whole transcriptome data in samples that passed robust regression and outlier removal methods (ROUTs) outlier elimination at weeks 20 and 29 was performed (all but 3 week-20 samples were found to be outliers and removed for PCA visualization) using the prcomp function in R. Differential gene expression between treatment arms and with continuous variables was determined by fitting a generalized linear model (GLM) to the transcript expression data using the edgeR package in R (74). The *P* values for the comparisons were corrected for multiple comparisons using the Benjamini-Hochberg (BH) method. Preranked GSEA (38) with 1000 permutations were done using MSigDB’s Hallmark/Reactome module (40), selected c7 module gene sets (43) (GEO GSE40666 and GSE15930), restriction factor gene set, and immune cell subset signatures (44). Preranking was done on all genes in decreasing order of the “(negative)log_10_*P*.value*log_2_FC” (used for group comparison between anti–IFN-α-treated and control groups) or correlation coefficient value (change in LN vDNA or plasma cytokines). After running GSEA, normalized enrichment scores and *P* values were obtained (adjusted further using the BH method). Pathways that passed an adjusted *P* value of less than 0.05 were considered significant. Overlapping leading-edge genes from different outcomes (where outcome 1 was anti–IFN-α vs. control and outcome 2 was change in LN vDNA) were tested for significant intersection using the Supertest function in R. The overlapping genes were then represented in heatmaps using the sample level enrichment analyses (SLEA) scores (75). Briefly, the *Z* score value of the overlapping gene set per sample (i.e., common genes from a pathway that is significantly upregulated by anti–IFN-α treatment and correlated with reduced LN vDNA) were calculated. The mean expression of significant genes as compared with expression of a set of 1000 random genes and the difference between observed and expected mean expression were calculated and represented on the heatmaps. Heatmaps were generated using the pheatmap package in R v4.0.3 (rows were clustered using Ward.D2 method with Euclidean distance) or using GraphPad Prism 9. A 3D plot was generated using the “car” and “rgl” packages in R v4.0.3. All correlation networks were generated in Cytoscape v3.8.2, and the Circos plot was generated using the online platform provided by http://mkweb.bcgsc.ca

### Statistics.

Wilcoxon’s rank-sum paired test was used to assess significant differences between 2 groups of paired samples (*P* < 0.05 was considered significant). Similarly, the Mann-Whitney *U* test was used to assess all other group differences (*P* < 0.05 was considered significant). Unless indicated, 2-group comparisons were visualized using violin plots (with each sample indicated by dots), and median and 25th and 75th percentiles were defined by dotted lines within the violins. Correlation analyses were performed using the nonparametric Spearman’s test (*P* < 0.05 was considered significant). *P* values for all univariate analyses and ρ values of Spearman’s correlations are shown on each figure. Univariate analyses were done in GraphPad Prism 9 or R v4.0.3.

### Study approval.

All protocols were approved before implementation by the IACUC at Bioqual Inc. (Rockville, Maryland, USA). Health, food intake, and body weights were recorded regularly according to established protocols.

## Author contributions

This manuscript has two co–first authors: LAS and AAS. LAS is listed as the first of the two co–first authors as she was responsible for conceptualizing the study and played a key role in project management, conducting experiments, data acquisition/analyses, and manuscript writing/submission. AAS is listed as the second co–first author of this study and was largely involved in big data analyses/modeling, data management, immunological interpretation of the findings, and manuscript writing. KG analyzed data. SPR conducted mesoscale experiments and acquired data. PW analyzed data. RMD designed research studies. RGA conducted experiments and acquired data. SW conducted experiments and acquired data. JDE conducted experiments and acquired and analyzed data. MP conducted experiments and acquired data. SGD provided scientific guidance. PWH provided scientific guidance. RPS provided scientific guidance. JMM designed research studies and provided scientific guidance

## Supplementary Material

Supplemental data

Supplemental tables 1-8

## Version 1 02/01/2022

In-Press Preview

## Version 2 03/08/2022

Electronic publication
