# Supplementary material for: IFN-α blockade during ART-treated SIV infection lowers tissue vDNA, rescues immune function, and improves overall health
Source: JCI Insight. 2022 Mar 8;7(5):e153046. doi: 10.1172/jci.insight.153046 (PMC8983135; doi:10.1172/jci.insight.153046)
Supplement: Supplemental data [file jciinsight-7-153046-s053.pdf]

Supplementary Data

Title

IFNα-blockade during ART-treated SIV infection lowers tissue vDNA, rescues immune function, and improves overall health

Supplementary Figure 1. Anti-IFNα treatment and the associated decline in LN vDNA is seen in conjunction with down-regulation of pDC/TI-IFN genesets. A. LN vDNA (normalized to LN CD4+ T cell frequency) in control and anti-IFNα treated arms during ART (i.e., week 20-36). **B**. Lack of association in LN vDNA levels and incomplete plasma viral suppression during ART. **C**. Reduced IFNα-inducible genes post-anti-IFNα treatment (i.e., lower expression of Hallmark TI-IFN geneset in **Figure 1F**) is characterized by a downregulation of master regulators of TI-IFN responses, IRF7 (upper plot) and IRF9 (lower plot) (p-values computed using Mann-Whitney U-test) (**Table S3**). **D.** Heatmap showing the sample level leading-edge scores of immune/metabolic genesets (extracted from MSigDB's Hallmark module) and cell subset signatures significantly altered with anti-IFNα treatment (compared to the control arm; week 29) and/or associated with decline in LN vDNA (column annotations shown in purple) (**Table S3**). Gene Set Enrichment Analysis (GSEA) was used to determine significance with each outcome and significant associations (p-value < 0.05) were marked (*). Sample Level Enrichment Analyses (SLEA) intersecting leading-edge genes (obtained from GSEA results against both outcomes) are shown in the heatmap.

Supplementary Figure 2. Induction of pro-inflammatory genesets post-IFNα blockade in rectal biopsies and whole blood. A-B. Leading edge genes in pDC and B-cell signatures (i.e., genes identified post-GSEA – Figure 1F - that define significant differences in cell subset genesets post-anti-IFNα treatment or that showed significant association with LN vDNA). Only the genes that are members of ReactomeDB Immune System sub-category are represented in the networks. Blue node denotes leading-edge genes that are members of pathways downregulated post-anti-IFN treatment or associated with higher vDNA levels. **C-D.** Leading edge genes in mDC and monocyte signatures (i.e., genes identified post-GSEA – **Supplementary Figure 1D** - that define significant differences in cell subset genesets post-anti-IFNα treatment or that showed significant association with LN vDNA). Only the genes that are members of ReactomeDB Immune System sub-category are represented in the networks. Red nodes indicate leading-edge genes that are members of pathways upregulated post-anti-IFNα treatment or associated with lower vDNA. **E.** Leading-edge genes from two significantly altered genesets (Hallmark's Type I IFN response was reduced and TNFα via NF-κB signaling geneset was induced) in rectal biopsies 9 weeks post-IFNα blockade. Gene Set Enrichment Analysis (GSEA) was used to determine significance with each outcome and significant associations (p-value < 0.05) were marked(*). Sample Level Enrichment

Analyses (SLEA) intersecting leading-edge genes (obtained from GSEA results against both outcomes) are shown in the heatmap.

Supplementary Figure 3. Anti-IFNα treatment and the associated decline in LN vDNA are correlated with increased expression of transcription factors (and targets) responsible for T cell proliferation/differentiation, and with decreased expression of transcription factors (and targets) that drive T cell quiescence and stemness cascades. A. Heatmap showing leading-edge genes that define the association of the decline in LN vDNA with expression of transcription factor targets that regulate T cell effector function (i.e., increased STAT5A targets and reduced TCF7 targets). Targets were extracted from CHEA and ENCODE databases and enrichment was assessed using GSEA (p-value<0.05; see Table S4 for details). **B.** Correlation networks showing the interaction between gene-sets associated with decline in LN vDNA (SLEA scores computed from leading-edge genes post-GSEA; GSEA p-value<0.05), C-**D.** Cytokine Clusters 4/5 **(C)** and effector CD8 T cell signatures **(D)** identified in Figure 3A. Edges represent correlations (Spearman's rho; p-value<0.05) and node color represents change in SLEA scores between anti-IFNα treated and untreated groups. **E.** Transcription factors (with known targets extracted from CHEA and ENCODE databases) that drive effector differentiation of T cells (i.e., STAT4, ID2) were associated with a decline in plasma TGFβ1, TGFβ2, and LN vDNA levels at week 29. Correlation network edges based on rho values obtained from Spearman's correlation test (p-value<0.05).

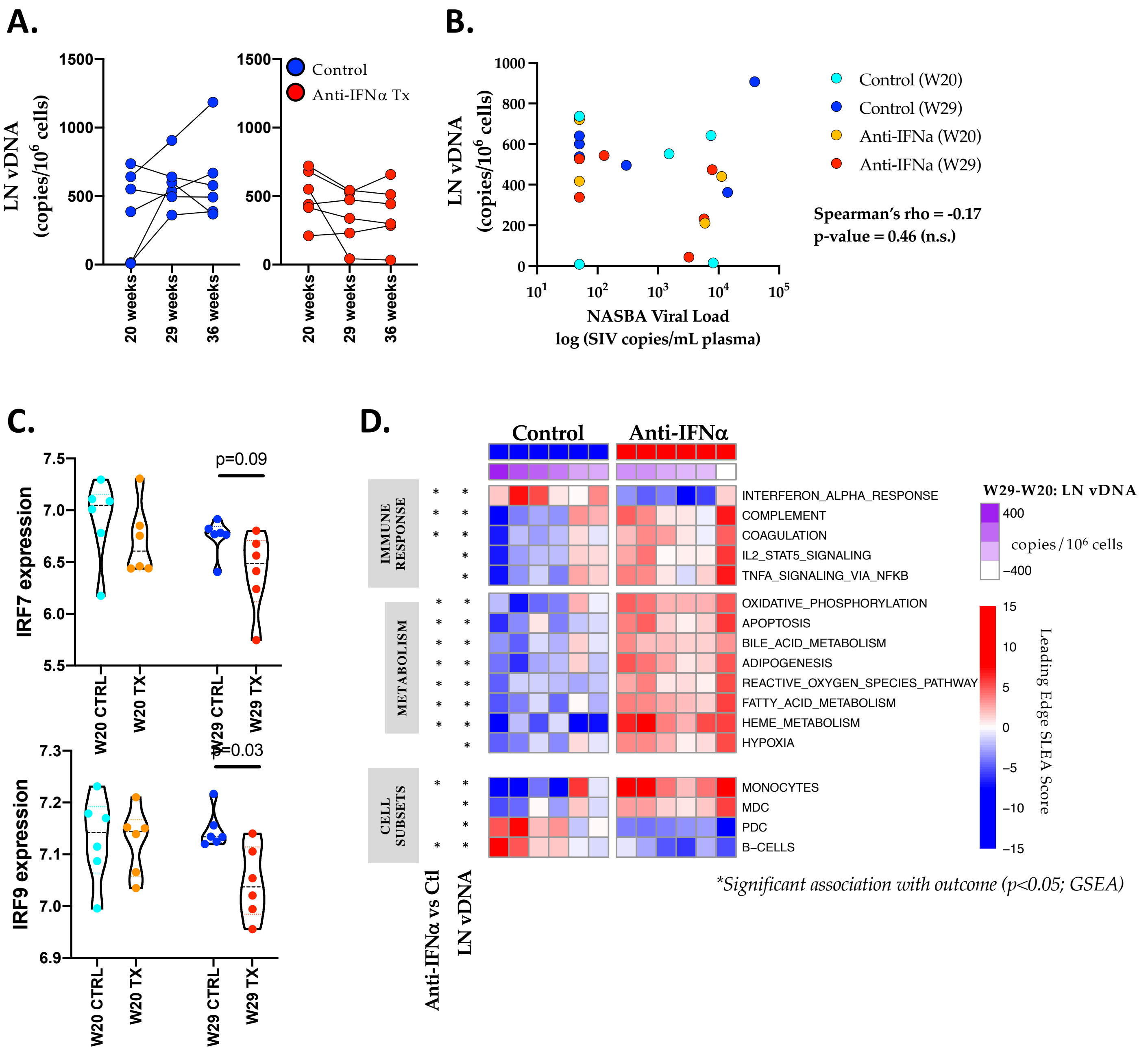
A.
LN vDNA (copies/10^6 cells)
1500
1000
500
0
20 weeks
29 weeks
36 weeks
Control
Anti-IFNα Tx
B.
LN vDNA (copies/10^6 cells)
1000
800
600
400
200
0
10^1
10^2
10^3
10^4
10^5
NASBA Viral Load
log (SIV copies/mL plasma)
Control (W20)
Control (W29)
Anti-IFNa (W20)
Anti-IFNa (W29)
Spearman's rho = -0.17
p-value = 0.46 (n.s.)
C.
IRF7 expression
7.5
7.0
6.5
6.0
5.5
p=0.09
W20 CTRL
W20 TX
W29 CTRL
W29 TX
IRF9 expression
7.3
7.2
7.1
7.0
6.9
p=0.03
W20 CTRL
W20 TX
W29 CTRL
W29 TX
D.
Control
Anti-IFNα
IMMUNE RESPONSE
INTERFERON_ALPHA_RESPONSE
COMPLEMENT
COAGULATION
IL2_STAT5_SIGNALING
TNFA_SIGNALING_VIA_NFKB
METABOLISM
OXIDATIVE_PHOSPHORYLATION
APOPTOSIS
BILE_ACID_METABOLISM
ADIPOGENESIS
REACTIVE_OXYGEN_SPECIES_PATHWAY
FATTY_ACID_METABOLISM
HEME_METABOLISM
HYPOXIA
CELL SUBSETS
MONOCYTES
MDC
PDC
B-CELLS
W29-W20: LN vDNA
400
copies / 10^6 cells
−400
15
10
5
0
−5
−10
−15
Leading Edge SLEA Score
*Significant association with outcome (p<0.05; GSEA)
Anti-IFNα vs Ctl
LN vDNA

Supp Fig. 1

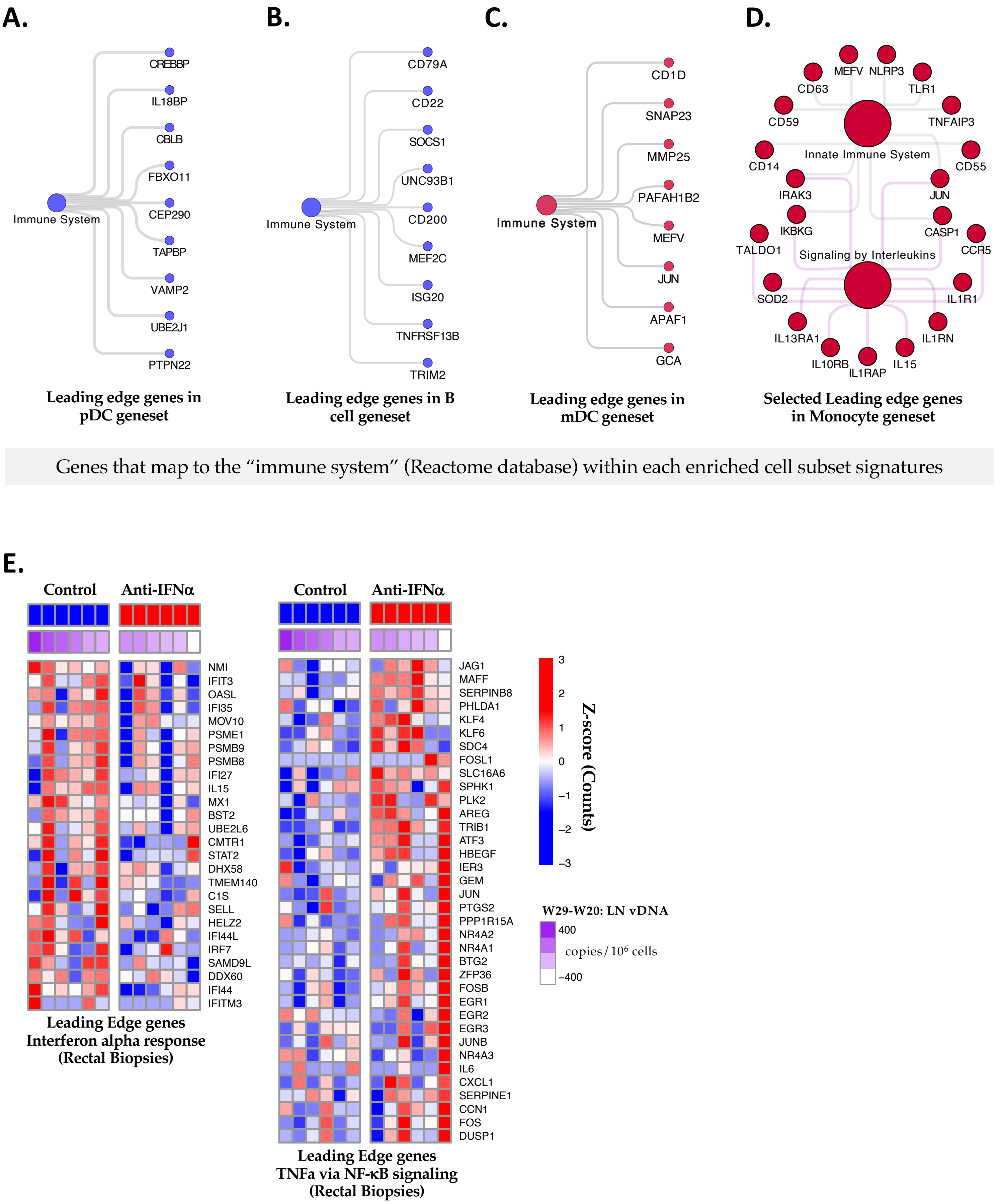
A.
Immune System
CREBBP
IL18BP
CBLB
FBXO11
CEP290
TAPBP
VAMP2
UBE2J1
PTPN22
Leading edge genes in pDC geneset
B.
Immune System
CD79A
CD22
SOCS1
UNC93B1
CD200
MEF2C
ISG20
TNFRSF13B
TRIM2
Leading edge genes in B cell geneset
C.
Immune System
CD1D
SNAP23
MMP25
PAFAH1B2
MEFV
JUN
APAF1
GCA
Leading edge genes in mDC geneset
D.
Innate Immune System
Signaling by Interleukins
MEFV
NLRP3
CD63
TLR1
CD59
TNFAIP3
CD14
CD55
IRAK3
JUN
IKBKG
CASP1
TALDO1
CCR5
SOD2
IL1R1
IL13RA1
IL1RN
IL10RB
IL1RAP
IL15
Selected Leading edge genes in Monocyte geneset
Genes that map to the "immune system" (Reactome database) within each enriched cell subset signatures
E.
Control
Anti-IFNα
NMI
IFIT3
OASL
IFI35
MOV10
PSME1
PSMB9
PSMB8
IFI27
IL15
MX1
BST2
UBE2L6
CMTR1
STAT2
DHX58
TMEM140
C1S
SELL
HELZ2
IFI44L
IRF7
SAMD9L
DDX60
IFI44
IFITM3
Leading Edge genes Interferon alpha response (Rectal Biopsies)
Control
Anti-IFNα
JAG1
MAFF
SERPINB8
PHLDA1
KLF4
KLF6
SDC4
FOSL1
SLC16A6
SPHK1
PLK2
AREG
TRIB1
ATF3
HBEGF
IER3
GEM
JUN
PTGS2
PPP1R15A
NR4A2
NR4A1
BTG2
ZFP36
FOSB
EGR1
EGR2
EGR3
JUNB
NR4A3
IL6
CXCL1
SERPINE1
CCN1
FOS
DUSP1
Leading Edge genes TNFa via NF-κB signaling (Rectal Biopsies)
Z-score (Counts)
3
2
1
0
−1
−2
−3
W29-W20: LN vDNA
400
copies/10⁶ cells
−400

Supp Fig. 2

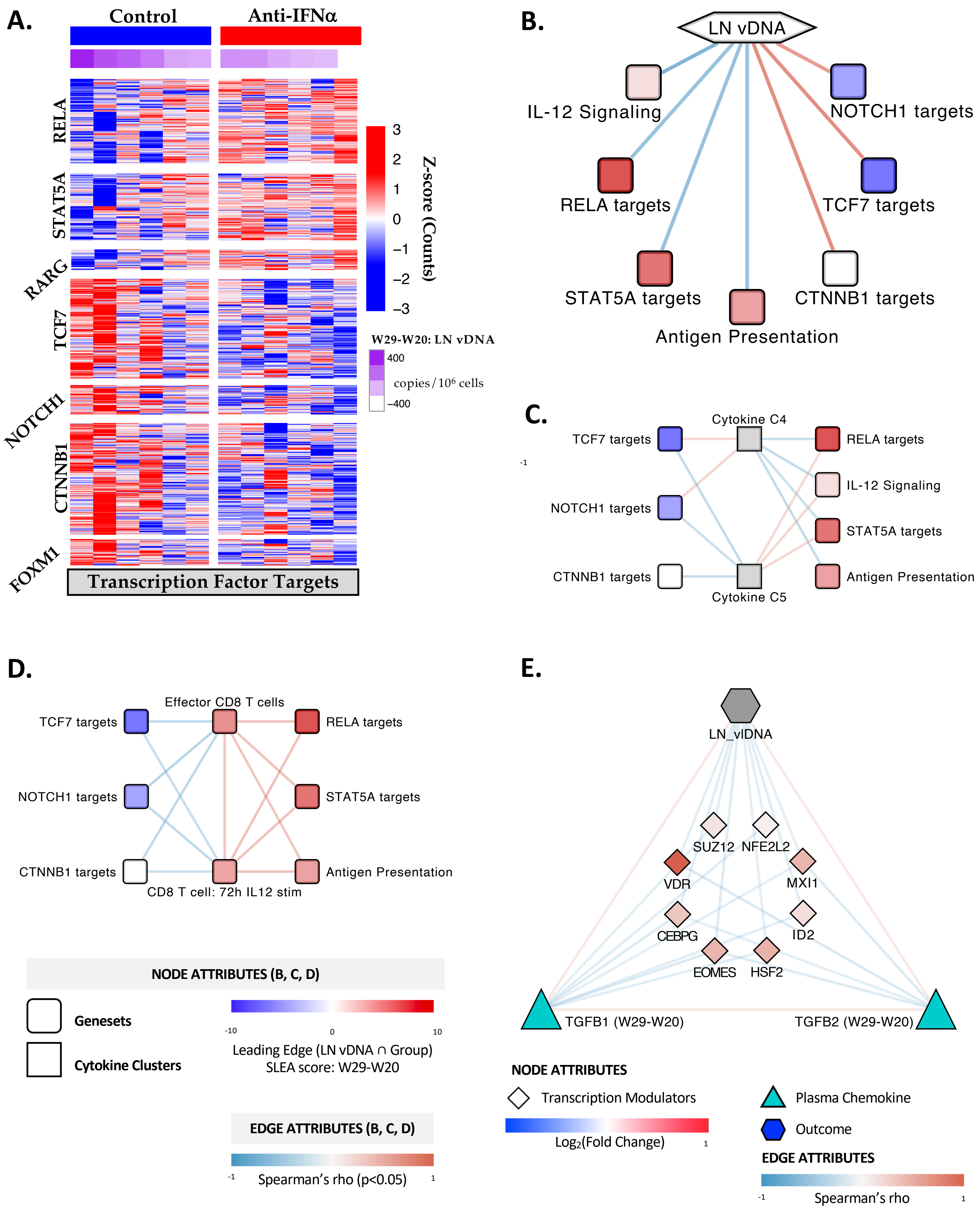
A.
Control
Anti-IFNα
RELA
STAT5A
RARG
TCF7
NOTCH1
CTNNB1
FOXM1
Transcription Factor Targets
Z-score (Counts)
3
2
1
0
−1
−2
−3
W29-W20: LN vDNA
400
copies / 10⁶ cells
−400
B.
LN vDNA
IL-12 Signaling
NOTCH1 targets
RELA targets
TCF7 targets
STAT5A targets
CTNNB1 targets
Antigen Presentation
C.
Cytokine C4
TCF7 targets
RELA targets
IL-12 Signaling
NOTCH1 targets
STAT5A targets
CTNNB1 targets
Antigen Presentation
Cytokine C5
D.
Effector CD8 T cells
TCF7 targets
RELA targets
NOTCH1 targets
STAT5A targets
CTNNB1 targets
Antigen Presentation
CD8 T cell: 72h IL12 stim
NODE ATTRIBUTES (B, C, D)
Genesets
Cytokine Clusters
-10
0
10
Leading Edge (LN vDNA ∩ Group)
SLEA score: W29-W20
EDGE ATTRIBUTES (B, C, D)
-1
Spearman's rho (p<0.05)
1
E.
LN_vDNA
SUZ12
NFE2L2
VDR
MXI1
CEBPG
ID2
EOMES
HSF2
TGFB1 (W29-W20)
TGFB2 (W29-W20)
NODE ATTRIBUTES
Transcription Modulators
Plasma Chemokine
Log₂(Fold Change)
1
Outcome
EDGE ATTRIBUTES
-1
Spearman's rho
1

Supp Fig. 3
